# Advances and Challenges in Minimally Invasive Myomectomy: A Narrative Review

**DOI:** 10.3390/jcm14124313

**Published:** 2025-06-17

**Authors:** Pengfei Wang, Noemi J. Hughes, Alireza Mehdizadeh, Camran Nezhat, Farr Nezhat

**Affiliations:** 1 Department of OB/GYN, BronxCare Health System, Bronx, NY 10457, USA; pwang@bronxcare.org (P.W.); nhughes@bronxcare.org (N.J.H.); amehdiza@bronxcare.org (A.M.); 2 Obstetrics, Gynecology and Reproductive Science, Icahn School of Medicine at Mount Sinai, New York, NY 10025, USA; 3 Department of OB/GYN, Stanford University Medical Center, Palo Alto, CA 94305, USA; camran@camrannezhatinstitute.com; 4 Department of OB/GYN, University of California San Francisco, San Francisco, CA 94143, USA; 5 Camran Nezhat Institute, Center for Special Minimally Invasive and Robotic Surgery, Woodside, CA 94061, USA; 6 Nezhat Surgery for Gynecology/Oncology, Valley Stream, NY 10128, USA; 7 Department of OB/GYN, Weil Cornell Medical College of Cornell University, New York, NY 10065, USA; 8 Department of OB/GYN, NYU Long Island School of Medicine, Mineola, NY 11501, USA

**Keywords:** myomectomy, minimally invasive surgery, laparoscopy, robot, LAM

## Abstract

Uterine fibroid is one of the most common benign uterine diseases, affecting up to 70–80% of females of reproductive age. Whilst abdominal myomectomy has traditionally been a major uterine-sparing surgical intervention for its management, this is not without considerable technical challenges and the potential for multiple complications and morbidity. Since the introduction of video-assisted endoscopic surgery by Dr. Camran Nezhat in the 1980s, the development of minimally invasive approaches to myomectomy has accelerated rapidly worldwide. Whilst this offers numerous benefits for patients, laparoscopic myomectomy still carries implications for necessary expertise in surgical skill, intraoperative hemorrhage control, concern for future reproductive potential and risk of occult uterine malignancy. In this review article, we present the latest data regarding such aspects and offer our opinions on widely raised questions and existing contentions regarding myomectomy. We believe that minimally invasive myomectomy is a safe, efficient and beneficial approach to management in the hands of surgeons empowered with advanced knowledge, experience, and refined surgical skills.

## 1. Introduction

**Figure 1:**
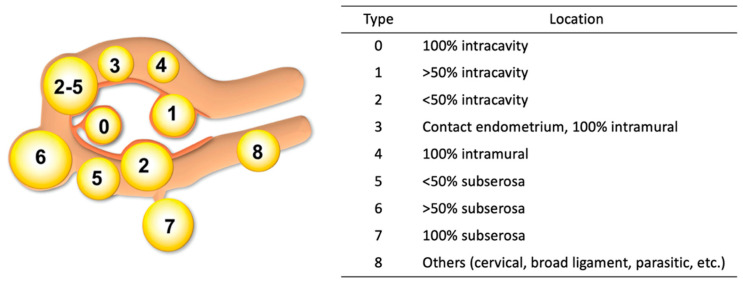
FIGO classification of uterine fibroids.

Uterine leiomyoma, or fibroid, is the most common benign tumor in women. The estimated cumulative incidence reported is up to 70% in white women and approaches 80% in black women during the premenopausal years [1]. In 2011, the Federation International of Gynecology and Obstetrics (FIGO) published a classification system for categorizing the location of fibroids [2]. Based on the location in relation to the endometrial and serosal surface, FIGO classifies fibroids from type 0 to type 8 (Figure 1). Though imperfect, this new classification has significantly facilitated research and recommendations for the clinical management of fibroids through international unification of the nomenclature. The original classification of fibroids can be traced back to work by Dr. Wamsteker et al. in 1993 [3]. This group developed a system for classifying submucosal fibroids based on their relationship to the endometrial surface with the intent to evaluate the challenges presented by and guide the practice of hysteroscopic myomectomy. FIGO has since adopted and extended this classification to all fibroids in the uterus, describing their relationship to both the serosal and mucosal surfaces. It should be noted, however, that determination of FIGO class is largely based on pelvic imaging with transvaginal ultrasound or magnetic resonance imaging (MRI) and interpretation of such images can be highly subjective. This was demonstrated by the study to evaluate the reproducibility of uterine fibroids among four providers, using the FIOG staging system. Surprisingly, only 14% of fibroids had a unanimous classification agreement. A total of 86% of cases had at least two unique answers, and 10% of fibroids had four unique classifications [4]. Thus, it is recommended that a gynecologic surgeon personally review a patient’s images and not solely rely on radiological reporting.

Most women with fibroids are asymptomatic; however, 15% to 30% can develop severe symptoms such as heavy menstrual bleeding, bulk symptoms, pelvic pain, infertility, or adverse pregnancy outcomes [5,6,7]. The liberal referral for imaging studies in the current medical culture has demonstrated more women with asymptomatic fibroids, who are often directed towards a specialist OB/GYN provider. This is an unfortunate consequence of the potential inexperience of primary care physicians in the management of fibroids as well as the increasing inclination of providers to practice defensive medicine. Symptomatic women do, however, require management, and the cost burden of this in the United States is reported to be USD 5.9 to USD 34.4 billion every year [8]. Symptomatic fibroid is the primary indication for hysterectomy in the United States and accounts for more than 400,000 cases of this surgery annually [9]. Alternative modalities frequently used for management include medication [10,11,12,13,14], radiofrequency ablation [15], uterine artery embolization [16,17,18], and myomectomy.

For the purposes of preserving childbearing potential, myomectomy is a surgical option for management which has been adopted since 1931. In contrast to hysterectomy, the initial steps of ligating all major blood vessels are avoided in myomectomy to not compromise fertility. In light of this, myomectomy is generally considered a more challenging procedure compared to hysterectomy. Historically, open surgery was adopted as the standard approach by all specialties under the “big surgeon, big incision” paradigm. It was in the late 1970s when Dr. C. Nezhat connected an old-fashioned camera to a monitor and in doing so freed a surgeon’s hands, eyes and feet, and a new era of minimally invasive surgery (MIS) was born. “Operative endoscopy will replace almost all open procedures” was a groundbreaking statement made by Dr. Nezhat in 2004 [19] which has since been realized. In current practice, there is rarely a debate as to whether an open approach should be adopted in place of MIS regardless of specialty. Dr. Semm was the first to report his experience of laparoscopic myomectomy in 1979 [20]. Although it remains a complex procedure, it was rapidly adopted by gynecologic surgeons worldwide due to the many benefits conveyed by MIS for patients. The advancement of instruments and technologies over the past 40 years has greatly facilitated the development and safety of laparoscopic myomectomy; however, new challenges continue to be encountered. In general, myomectomy is considered a challenging operation, which may become even more foreboding to gynecologists if the approach is to be in a minimally invasive fashion. Thus, there is an ever-increasing and critical need for research into its safe and feasible methodology. Furthermore, the debate surrounding the role of myomectomy in the treatment of infertility is ongoing; however, more convincing data appear to emerge as there is increasing research into the field of IVF (in vitro fertilization). Similarly, more data are available regarding obstetric outcomes following myomectomy, in particular minimally invasive myomectomy. Lastly, the possibility of occult uterine sarcoma remains a heated discussion with largely inconsistent conclusions since the FDA ban on clinical use of power morcellation in the USA in 2014 [21]. In this narrative review, we present and discuss the most recent literature published on minimally invasive myomectomy and focus on these three topics. We hope that the conclusions may shed new light on this clinical practice and encourage more gynecologic surgeons to perform minimally invasive myomectomy successfully and comfortably.

## 2. Clinical Features

### 2.1. Abnormal Uterine Bleeding

The duration of normal menstrual bleeding is typically five days occurring at a cycle between 21 and 35 days. Abnormal uterine bleeding (AUB) is the terminology used to encompass menstrual bleeding which falls outside of this pattern, including menorrhagia, metrorrhagia, polymenorrhagia, and oligomenorrhea. Menorrhagia, or heavy menstrual bleeding, is defined as menstrual blood loss exceeding 80 mL or bleeding which persists for more than eight days [22]. AUB is considered chronic when it has occurred for at least six months, or acute when an episode of heavy bleeding warrants immediate intervention. Chronic heavy bleeding is the most common symptom resulting from fibroids; however, it is not uncommon for affected women to present to the emergency room with an acute on chronic episode of extremely heavy bleeding [23,24]. Such excessive bleeding can significantly impact a patient’s quality of life, both medically if it precipitates secondary conditions such as iron deficiency anemia and psychosocially if it interferes with functioning in their work or personal matters [24].

The exact etiology of AUB associated with fibroids remains unclear. Several groups have proposed a variety of theories including obstruction from fibroids leading to development of venule ectasia in the endometrium [25], increased surface area of the endometrium [26], dysregulation of local growth factors and aberrant angiogenesis within the uterus and endometrium [27], platelet action overcome by vascular flow in engorged vessels [27], increased uterine contractility and peristalsis [28,29,30,31], and alterations to the vasoconstriction of spiral aterioles [29]. The ultimate outcome of AUB from fibroids might be a combination of all aforementioned factors, since multiple different directions of intervention such as increasing coagulability, inducing hypoestrogenism, and removal of fibroids seem to have a role in its management. It should be emphasized that the diagnosis of AUB is also more complicated than the given nomenclature. The counting of pads or cups to quantify menstrual blood loss is not reliable and has steered influential bodies such as the Royal College of Obstetricians and Gynaecologists (RCOG) and the American College of Obstetricians and Gynecologists (ACOG) towards the more patient-centered definition of AUB as “excessive menstrual blood loss which interferes with patients’ physical, social, emotional and or material quality of life” [32]. The prevalence of AUB is significant and reported to be between 10% and 30% among women of reproductive age [33]. The importance of questioning the amount and duration of menstrual bleeding during a patient’s annual exam is also exemplified in a study by Nelson et al. [23]. In a group of 149 women with severe anemia (defined as a hemoglobin < 5 g/dL), 90.4% women reported chronic excessive blood loss; however, 33.9% were discharged without offer of therapy to prevent subsequent bleeding, 35.1% were lost to follow-up prior to receiving effective therapy, and 26.8% received multiple transfusions before seeking or receiving definitive treatment [23].

### 2.2. Bulk Symptoms

Mass effects secondary to fibroids such as pelvic pressure, urinary frequency, and constipation are collectively referred to as bulk symptoms [34]. Although less common, more severe bulk symptoms also include hydronephrosis and compressive neuropathy [35]. Bulk symptoms from fibroids can detrimentally affect a patient’s quality of life; however, studies on this topic are limited. COMPARE-UF is a multicenter prospective study evaluating the outcomes of the treatment of fibroids via different modalities [36]. It showed that 74.2% of women seeking medical management reported bulk symptoms [37,38]. Urinary symptoms, especially voiding disorders, in women with fibroids have been severely underestimated and less frequently studied. According to one study by Vecchioli-Scaldazza et al., 72.8% of women who were hospitalized for fibroids had urinary symptoms. Following management with hysterectomy or myomectomy, 87.5% of women were asymptomatic or experienced resolved symptoms. This improvement was also demonstrated in urodynamic parameters [39]. The COMPARE-UF study found that women experienced a significant improvement in their total health-related quality of life and symptom severity scores from baseline at one year follow-up. The greatest improvements were seen across all treatment modalities including abdominal hysterectomy, laparoscopic hysterectomy, abdominal myomectomy, laparoscopic myomectomy, and uterine artery embolization [37].

### 2.3. Infertility

In theory, fibroids can affect fertility, and the relationship between fibroids and infertility or subfertility has been a topic of research extending over decades. To begin with, fibroids can disrupt physiological myometrial motility and interfere with sperm progression and embryonic implantation [30,31,40]. They can subvert the pelvic anatomy, thereby perturbing the function of the fallopian tubes [41]. Finally, submucosal and possibly intramural lesions can create an inflammatory endometrial milieu deleteriously affecting sperm migration and embryonic implantation [42]. The hypotheses of detrimental effects of fibroids on fertility were collectively reviewed by Dr. Don in 2023 [43]; however, epidemiological studies on this matter remain controversial. This is largely owing to the complexity of the etiology of infertility and the heterogeneity of fibroids confound the methodology of many studies [44]. The development and popularization of in vitro fertility (IVF) treatment have led to more robust data and conclusions on the matter; however, it remains heavily subject to debate.

Fibroids are the sole reason accounting for 2% to 3% of cases of infertility. It has been demonstrated that submucosal fibroids or those deforming the uterine cavity decrease pregnancy, whilst subserosal fibroids have no apparent detrimental effect on fertility [6,43,45,46,47,48]. The most recent meta-analysis from Pritts et al. showed the relative risk of ongoing pregnancy and delivery rate associated with submucosal fibroids to be 0.36 (95% CI 0.18 to 0.74) and 0.32 (95% CI 0.12 to 0.85), respectively [6]. Furthermore, multiple studies have shown that the removal of submucosal fibroids increases the rate of pregnancy and live birth rate for both spontaneous pregnancies and those resulting from IVF [49,50]. The effect of intramural fibroids without uterine cavity distortion remains contested, with conflicting conclusions from different studies. Whilst historically it was believed that they do not affect fertility or conception and there is no improvement in fertility with the removal of noncavity-distorting intramural fibroids [43,51,52], there is emerging evidence indicating that intramural fibroids exceeding a certain size may have negative effects at least in IVF. The latest meta-analysis of five studies compared 520 women with type 3 fibroids to 1392 women without fibroids undergoing IVF. The primary outcome measure was lower birth rate and the secondary outcome measures were clinical pregnancy, implantation, and miscarriage rates. The data showed that women with non-cavity-distorting intramural fibroids ≤ 6 cm had a significantly lower birth rate compared to women with no fibroids (OR: 0.48, 95% CI: 0.36–0.65). A significant reduction in low birth rate was also noted in the group with ≤4 cm fibroids, but not in the ≤2 cm fibroid group. Therefore, 2–6 cm sized noncavity-distorting intramural fibroids have a deleterious effect on LBR (live birth rate) in IVF [53]. Crucially, another study also demonstrated a statistically significant association between an increased number or larger size of fibroids and poorer IVF outcomes [54]. Considering this controversy and the possible complications associated with myomectomy, shared decision-making for management should be adopted following adequate counselling of patients, especially for IVF cases.

## 3. Laparoscopic Myomectomy

Since Dr. Reich performed the first laparoscopic hysterectomy in 1989, cases of laparoscopic and robotic hysterectomy have increased dramatically worldwide. Per the recent literature, the proportion of laparoscopic hysterectomies is reported to have increased from 26.1% to 43.4%, with a concomitant decrease in the proportion of abdominal (38.6% to 28.3%), laparoscopic-assisted vaginal (20.2 to 16.7%) and vaginal (15.1% to 11.5%) hysterectomies in the USA between 2010 and 2013 [55]. In 2018, minimally invasive hysterectomy accounted for 65.8% of hysterectomy cases [56]. Internationally, a report from Turkey showed the ratio of laparoscopic total hysterectomy to all hysterectomies performed at one institute increased from 2.4% in 1995 to 44.7% in 2018—a rate 33 times higher over 24 years [57]. The situation with laparoscopic myomectomy, however, differs greatly from that of hysterectomy. In a recent cross-sectional study of 114,850 myomectomy cases using the National Inpatient Sample database between 1 January 2010 and 31 December 2014 [58], 330 (7%) were reported to be minimally invasive whilst 106,520 (93%) were open. Over time, the proportion of minimally invasive myomectomy cases has remained very low and even decreased from 8.2% in 2010 to 6.1% in 2014. Most hospitals document few minimally invasive myomectomy procedures per year, with 50% performing five or fewer and 25% performing three or fewer per year. In addition, the study found African American, Hispanic, and women of other races were less likely to undergo minimally invasive myomectomy compared to Caucasian women [58]. A different study from the Vancouver Coastal Health and Providence Health Care regions of British Columbia reported between 2007 and 2012 approximately 20% of myomectomies were performed laparoscopically and that this approach predominated at two of the five metropolitan area hospitals, suggesting a clustering of surgical skill at a few select institutes [59]. There is also an argument that the cost of minimally invasive myomectomy exceeds that of open surgery [60]. More data have indicated, however, that the overall cost of MIS is low owing to reduced operating time, length of hospital stay, and rate of readmission [61,62]. With the evolution of MIS expertise, same-day patient discharge following minimally invasive myomectomy has become both safe and readily feasible, leading to a considerable decrease in the overall cost of such surgery [63]. The critical limiting factor to widespread utilization of minimally invasive myomectomy, especially via laparoscopy, undoubtedly appears to be individualized surgeon skill. In order to achieve obstetric outcomes equal or similar to open myomectomy, several steps including judicious control of bleeding, adequate specimen retrieval, and myometrial closure must be assured, and this requires advanced skills and experience in MIS [64].

Compared to open myomectomy, minimally invasive myomectomy enables faster recovery for patients. One recent study found that women undergoing myomectomy reported substantial improvements in health-related quality of life, regardless of the route of surgery. Abdominal myomectomy was, however, associated with a close to two-week longer time to return to work compared to laparoscopic myomectomy [65]. A different large study of 66,012 women undergoing myomectomy reported that 5265 (8.0%) had at least one complication, and the minimally invasive approach was associated with a decreased risk of complications compared to open myomectomy (OR: 0.29; 95% confidence interval [CI], 0.25–0.33; *p* < 0.001) [66]. Our recent study also demonstrated some significant advantages of minimally invasive myomectomy in terms of the incidence of blood transfusion, intra-operative and post-operative complications, and better post-operative recovery. Crucially, marked benefits were observed in cases with technically challenging fibroid burden when myomectomy was performed using minimally invasive approaches [67].

## 4. Robotic Myomectomy

One of the most challenging skills in minimally invasive myomectomy is laparoscopic suturing. The robotic platform greatly facilitates this with the provision of impressive three-dimensional and magnified visualization, superior ergonomics and a natural, intuitive, dexterous control and manipulation of the surgical instruments [68]. As a result, minimally invasive myomectomy is becoming more popular since the introduction of robotic surgery in 2005. In a large retrospective cohort study of 4033 women undergoing myomectomy at Kaiser Permanents Northern California between 2009 and 2019, the rate of minimally invasive procedures grew from 6% to 89.5% [69]. This 15-fold increase occurred in parallel with a decline in the rate of abdominal myomectomy from 94.0% to 10.5% over the same period. The proportion of laparoscopic myomectomy cases increased from 6.0% to 24.9% and the rate of robotic myomectomy cases increased from 3.6% in 2012 to 64.6% in 2019 [69]. Such expansion of robotic surgery is staggering. According to another cohort study from the state of Michigan, the utilization of robotic surgery grew from 1.8% in 2012 to 15.1% in 2018. This was associated with a modest concomitant decrease in the rate of laparoscopic surgery from 53.2% to 51.3%. Before the adoption of robotic surgery, the rate of laparoscopic surgery at hospitals was reported to be increasing by 1.3% per year, which has since declined to 0.3% [70].

Compared to open myomectomy, the benefits of robotic myomectomy are significant and well demonstrated. One early meta-analysis from 2016 comprised eight studies comparing robotic to laparoscopic myomectomy and nine studies to the open myomectomy technique, including a total of 2027 women. Compared to open myomectomy, robotic surgery was associated with significantly longer operative time but much lower estimated blood loss, need for transfusion, total complication rate, and length of hospital stay [71]. The latest meta-analysis available includes 53 studies comparing laparoscopic, robotic, and abdominal myomectomy on outcomes of mean operative time, estimated blood loss, length of hospital stay, transfusion rate, complication rate, and rate of conversion to laparotomy. Robotic myomectomy was found to be superior to abdominal myomectomy in all parameters with the exception of mean operative time. It was also found to have similar outcomes to laparoscopic myomectomy with regard to overall estimated blood loss, length of hospital stay, transfusion rate, and complication rate. The robotic approach was associated with reduced estimated blood loss, observed in women with smaller fibroid burden, and a marginally lower rate of conversion to laparotomy. Laparoscopic myomectomy was found to be superior with regard to mean operative time and was the preferred option of approach in cases of larger fibroids [72].

It holds true that the overall outcomes of robotic and laparoscopic myomectomy are comparable in experienced surgeons’ hands [71,72]. The robotic technique, however, has been recognized to prolong mean operative time compared to laparoscopic myomectomy per Dr. Nezhat’s studies [73]. Regardless, the robotic platform offers an incomparable advantage for minimally invasive myomectomy owing to its shorter associated learning curve and the advances of technologies such as the Da Vinci Xi and robot 5, which enable easy docking and a more user-friendly platform to operate. Thus, we anticipate that more gynecology surgeons will begin to perform minimally invasive myomectomy, which appears superior to open surgery overall as above.

Data regarding the long-term outcomes of robotic myomectomy on fertility and pregnancy are rather limited. However, the available studies report similar results compared to laparoscopic myomectomy or open myomectomy in terms of fertility and pregnancy. Though the study sample was small, one Canadian cohort study demonstrated pregnancy rates following robotic myomectomy of 70% [74]. Another retrospective study reported a pregnancy rate of 80% following robotic myomectomy [75]. Compared to abdominal and laparoscopic myomectomy, the total ensuing medical costs of robotic myomectomy are significantly higher, largely secondary to the need for expensive equipment, its regular maintenance, and recurrent purchasing of specialized medical consumables [76,77]. One study centered on such cost analysis found abdominal myomectomy was the least expensive approach at USD 4937 compared with laparoscopic myomectomy at USD 6199 and robotic myomectomy at USD 7280 using a robot model. In their robot purchase model, the cost of robotic myomectomy increased incrementally by USD 2814, USD 1939, and USD 1090 per case when the amortized and maintenance costs were distributed over 12, 18, and 32 cases per month, respectively [60]. ACOG (the American College of Obstetrics and Gynecology) therefore recommends choosing to adopt robotic surgery based on the complexity of each case as well as patient factors, with appropriate consideration of the financial costs [78].

## 5. Laparoscopic-Assisted Myomectomy

Laparoscopic-assisted myomectomy (LAM) is an innovative surgical modality first introduced by Dr. Nezhat in 1994 [79]. The group initiated a paradigm where diagnostic laparoscopy was first performed to assess the fibroids, surrounding structures and possible adhesions. Following this, a Pfannenstiel mini-laparotomy of 2–3 cm was made and the fibroids were brought to the incision, morcellated with a scalpel. The myometrium was closed in a sequence of fibroid accessibility. Though it carries several advantages, this technique remains underutilized at present due to its technical challenges. However, we believe LAM has the potential to prevail as a modality for myomectomy due to its unique advantages.

The first advantage of LAM is the potential to diagnose and treat endometriosis concurrently with myomectomy. The primary indications for myomectomy are heavy menstrual bleeding, pelvic pain, and subfertility. Thus, it is not uncommon for there to be coexisting fibroids with endometriosis contributing to patient symptomatology. One retrospective study showed both fibroids and endometriosis were found in 21.2% of women during laparoscopic myomectomy. Endometriosis was more common in those with subfertility (44% vs. 25.7%; *p* = 0.02) and less common in those with bleeding disorders (20% vs. 45%; *p* = 0.003). Parity, location of fibroids, race, and ethnicity affected the risk of endometriosis, whereas size and number of fibroids did not [80]. The data from Dr. Nezhat are even more impressive, exemplified in a retrospective study conducted from March 2011 through December 2015. Of 208 women with a chief presenting complaint of symptomatic fibroids who underwent surgical intervention, 181 (87.1%) had both fibroids and endometriosis, whilst only 27 (12.9%) had fibroids alone. Of these 27 women, 9 were identified to have adenomyosis [81]. Originating from a renowned endometriosis center, there may be an element of selection bias in this data. Despite this, due to the increasing evidence of coexisting fibroids and endometriosis particularly in the subfertility population, the concomitant diagnosis and treatment of endometriosis via LAM is likely to benefit this group. Open myomectomy carries the risk of missing the diagnosis of endometriosis, since the disease is most likely to affect the posterior compartment of the pelvis.

Other advantages of LAM include the potential to evaluate for and treat adhesions and the benefits conveyed by a small incision. Mini-laparotomy for myomectomy without diagnostic or operative laparoscopy is reported to be associated with less pain and faster recovery for patients [82]. This approach does, however, increase the difficulty in accessing the uterus and limits exposure of the surrounding structures which can compromise the safety of the surgery. Due to the increasing incidence of cesarean section and other abdominal surgeries, it is not uncommon for gynecologists to encounter significant adhesive disease during myomectomy. Our recent study from a community hospital reported 31.5% of hysterectomy cases required concurrent extensive lysis of adhesions [83]. In light of this, initial diagnostic laparoscopy can provide information regarding the extent and location of adhesions so as to avoid complications during subsequent mini-laparotomy.

Compared to laparoscopic myomectomy, LAM permits the removal of a large burden of fibroid tissue. Regardless of surgeon skill, laparoscopic myomectomy entails longer operative time and the number of fibroids that can be removed is more limited. Using LAM, Dr. Nezhat performed myomectomies on uteri from eight to 26 weeks in size with excision of fibroids with a weight range 28 g to 998 g (mean 247 g) [79]. A different large data set from Dr. MacKoul included specimens from a total of 1313 conventional laparoscopic, robotic, LAM, and abdominal myomectomies performed between January 2011 and December 2013. The average number, size, and total weight of fibroids removed were comparable in the LAM and abdominal myomectomy groups (9.1, 8.13 cm, 391 g compared to 9.0, 7.5 cm, 424 g respectively; *p* < 0.001). Furthermore, the number and total weight of fibroids removed in these groups were significantly greater than those with conventional laparoscopic or robotic myomectomies (2.9, 217 g and 2.9, 269 g, respectively; *p* < 0.0001) [84]. We recently reported our experience of LAM procedure with the largest specimen of 3100 g [67]. With consideration of fibroid location, type 2 and 3 fibroids are not easily accessible via the laparoscopic approach, especially if small. Such fibroids may, however, distort the uterine cavity and account for heavy menstrual bleeding or subfertility. Their identification and removal largely rely on palpation by the operating surgeon, which can contribute to an extended operative time. Since patients are conventionally positioned in deep Trendelenburg for minimally invasive gynecology surgery, prolonged operative time carries the risk of adverse effects on cardiovascular, pulmonary, cerebral, and intraabdominal physiological function. The LAM procedure, however, with its benefits similar to open surgery, enables a surgeon to palpate type 2 and 3 fibroids and efficiently remove them.

**Figure 2:**
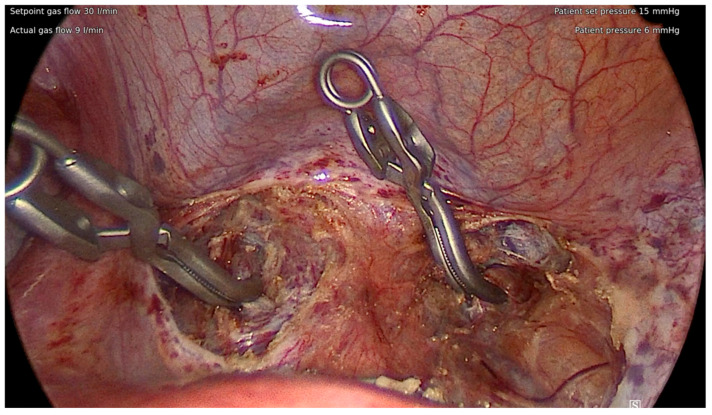
Temporary blockade of the uterine arteries at the anterior cul-de-sac.

The critical limitation of LAM lies in the potential difficulty in visualizing, accessing, and controlling intraoperative bleeding. Owing to the more restricted surgical field available and potential patient characteristics of multiple large fibroids not amenable to laparoscopic myomectomy, pharmacological methods to manage bleeding are usually insufficient. Accordingly, several groups have found a significantly higher intraoperative blood loss associated with LAM compared to laparoscopy alone [85]. Seidman et al., for example, used vasopressin to control bleeding in a study comparing LAM with laparoscopic and abdominal myomectomy. They found comparable weights of fibroids removed and intraoperative blood loss between the LAM and abdominal myomectomy groups; it should be noted that both outcomes were significantly lower in laparoscopic cases [86]. However, if the uterine artery can be blocked mechanically, the intraoperative bleeding will be controlled, and significant benefits of LAM procedures have been reported. Dr. MacKoul reported their experience of blocking the uterine arteries with a tourniquet at the isthmus of the uterus at the outset of LAM procedures and demonstrated excellent outcomes in an ambulatory surgical setting [87]. In addition to the removal of a fibroid burden similar to abdominal myomectomy with an intraoperative blood loss similar to laparoscopic myomectomy, this procedure was associated with minimal intraoperative and postoperative complications. The overall intraoperative and grade 3 postoperative complication rates were found to be 1.4% and 1.6%, respectively [84,88]. We proceeded to develop an alternative technique, in which the uterine arteries are dissected and temporarily occluded at the anterior cul-de-sac through a suprapubic incision (Figure 2). The same incision can be converted to a mini-laparotomy to complete myomectomy if the number or size of fibroids is too extreme and deemed unamenable to laparoscopic removal [67]. With the advent of such methodology to secure bleeding control, we believe LAM has the potential to become the preferred procedure for even complex myomectomy cases.

## 6. Long-Term Outcome of Minimally Invasive Myomectomy

The fertility-sparing advantage of myomectomy is inherently associated with a potential risk of further intervention or reoperation. Dr. Nezhat first reported in 1998 that the cumulative risk of such recurrence following laparoscopic myomectomy was 10.6% after 1 year, 31.7% after 3 years, and up to 51.4% after 5 years [89]. Dr. Yoo in 2007 reported similar results of the cumulative probability of recurrence following laparoscopic myomectomy at 11.7% after 1 year, 36.1% after 3 years, and 52.9% after 5 years [90]. There are, however, other studies reporting a far lower recurrence rate. Dr. Doridot, for example, found a cumulative recurrence risk of 12.7% at 2 years and 16.7% at 5 years [91]. Similarly, Dr. Radosa reported a cumulative recurrence rate of 4.9% at 2 years and 21.4% at 5 years [92]. It is exceedingly difficult to compare the results from such different studies due to the various definitions of recurrence used and the differing patient populations, including the number of fibroids in each case [91,92,93]. The majority of the literature, however, concurs that risk factors related to fibroid recurrence are fibroid number, size, patient age, the presence of associated pelvic disease, and preoperative use of GnRH agonist [90,93].

Although the reported recurrence rate varies significantly among different studies, most suggest the rate following laparoscopic myomectomy is higher compared to abdominal myomectomy. Hazard regression analyses used indicate a recurrence rate of up to 167% higher following a laparoscopic approach [94]. This is largely attributed to the increased difficulty of palpation and assessment of small residual fibroids with laparoscopy compared to laparotomy. The opportunity for manual removal of fibroids in abdominal myomectomy likely enables a more exhaustive extraction of even small fibroids, potentially leaving less residual disease [91,94]. A recent large-scale study involving 66.012 patients over 7.3 years does, however, present a very interesting result. Whilst the reoperation rates were higher in the minimally invasive myomectomy group over the entire study period (OR 2.33; 95% CI 1.95–2.79, *p* < 0.001), the odds of reoperation did decrease each year, with the odds of reoperation after abdominal myomectomy surpassing minimally invasive myomectomy in 2015. In addition, the odds of complications associated with minimally invasive myomectomy were found to decrease each year compared to abdominal myomectomy (OR 0.90; 95% CI 0.87–0.93, *p* < 0.001) [66]. Such results likely reflect the concurrent improvements in minimally invasive myomectomy techniques together with increased subspecialty training among surgeons [66].

Fibroid recurrence is one of the main problems associated with all uterine-sparing treatments for symptomatic fibroids. It must be noted, however, that only a small absolute portion of cases need reintervention. One meta-analysis reported a reintervention risk after 5 years of 12.2% for myomectomy, 14.4% for UAE (Uterine Artery Embolization), 53.9% for HIFU (High-Intensity Focused Ultrasound), and 7% for hysterectomy [95]. Dr. Doridot’s study did show a 22.9% recurrence rate after laparoscopic myomectomy, yet only 4.08% of patients required reintervention [91]. Similarly, Dr. Yoo reported a recurrence rate after laparoscopic myomectomy of 52.9% at 5 years and 84.4% at 8 years. the rate of reoperation was far lower with only 6.7% at 5 years and 16% at 8 years [90]. From the earliest experiences of Dr. Nezhat reporting on laparoscopic myomectomy, there were 38/114 (33.3%) recurrences after an average time interval of 27 months and 24 of 38 patients did not require retreatment [89]. Published literature surrounding fibroid recurrence following robotic myomectomy is more limited. One study from Dr. Pitter in 2015 investigating symptom recurrence after robotic myomectomy reported 81.3% of patients were symptom-free up to 12 months, 72% after 12–24 months, 64.8% after 24–36 months, and 62.9% at greater than 3 years from the index surgery [96].

Overall, the long-term outcomes from minimally invasive myomectomy seem highly promising, with ongoing improvements in surgical technology and the increased availability of surgeons with specialist training and expertise [66].

## 7. Advances in and Challenges of Minimally Invasive Myomectomy

### 7.1. Bleeding Control

Fibroids are highly vascular tumors, and the blood supply to the uterus can be significant, especially if there are multiple large fibroids. Regardless of the approach to myomectomy, one of the most common surgical complications is major hemorrhage, which can necessitate immediate intraoperative blood transfusion. Furthermore, life-threatening hemodynamic instability, shock, coagulopathy, and mortality are potential consequences if bleeding is not effectively controlled. According to one retrospective cohort study of 3407 cases of myomectomy, the overall rate of blood transfusion during or within 72 h of surgery was 10% (hysteroscopy 6.7%; laparoscopy 2.7%; abdominal 16.4%). Even with adjustment for confounding variables, women who required blood transfusions had an approximately threefold increased risk of major postoperative complications [97]. Thus, measures to minimize bleeding and associated complications are essential to lower the risk of morbidity and mortality in those undergoing myomectomy [98]. The currently adopted approaches to this can be summarized into medical and mechanical methods.

#### 7.1.1. Vasopressin

Intra-myometrial injection of vasopressin has been used routinely in myomectomy to reduce blood loss, though it has never been approved by the FDA (US Food and Drug Administration) for this indication. Vasopressin is a peptide hormone consisting of nine amino acids produced in the hypothalamus and the normal plasma concentration is less than 4 pg/mL [99]. There are three receptors for vasopressin, which are V1, V2, and V3. Whilst V1 and V2 receptors are distributed peripherally, V1 and V3 receptors are found in the central nervous system [100]. Owing to the expression of V1 receptors in the uterus, local injection of vasopressin can induce both vasoconstriction and uterine contraction [100]. A high concentration of vasopressin in the bloodstream can, however, produce adverse side effects such as bradycardia, pulmonary edema, myocardial ischemia, myocardial infarction, and cardiac arrest even in healthy patients [99,101,102,103,104]. Dr. Nezhat reported the first case of vasopressin-induced complications in laparoscopic myomectomy, and it has subsequently been recommended the drug is used judiciously with the minimal dose required to produce benefit [105]. The minimum concentration of vasopressin with therapeutic effect has been reported to be 0.05 U/mL and its half-life is only 10 to 35 min [100]. In light of this, it is unrealistic to rely solely on vasopressin to complete laparoscopic or robotic myomectomy with minimal bleeding, especially in cases of complex fibroids. Additional methods should therefore be used in combination with vasopressin to create a synergistic effect to minimize bleeding.

#### 7.1.2. GnRH Agonists and Antagonists

The use of gonadotropin hormone-releasing hormone (GnRH) agonists is approved by the FDA for the medical treatment of menorrhagia and as a bridge to definitive surgical management of fibroids. Treatment with a GnRH agonist can increase hemoglobin levels as well as decrease the size and volume of fibroids. A recent meta-analysis showed that pretreatment with GnRH agonists prior to laparoscopic myomectomy decreases intraoperative blood loss by 97.37 mL compared to no pretreatment or placebo. The administration of a GnRH agonist before laparoscopic myomectomy was also associated with a reduced frequency of blood transfusion compared to no pretreatment [106]. It must be noted, however, that use of GnRH agonists can increase the difficulty of myomectomy by complicating identification of cleavage planes between the uterus and fibroids, in turn increasing the time required for their nucleation [106]. In the same meta-analysis, the risk of fibroid recurrence was found to be significantly higher following pretreatment with GnRH agonists compared to no pretreatment or placebo in laparoscopic myomectomy and the main reason accounting for this is likely the incomplete removal of fibroids owing to the loss of clear surgical planes [106]. Thus, the indication for pretreatment with GnRH agonists should be evaluated on a case-by-case basis with consideration of the risks versus benefits. We generally avoid GnRH agonists in the immediate presurgical planning for myomectomy.

GnRH antagonists are a group of newly emerging medications which demonstrate similar or better effects on fibroids, adenomyosis, and endometriosis compared to GnRH agonists. Elagolix and Relugolix are the two FDA-approved GnRH antagonists currently available on the market [107]. To date, the effects of their utilization prior to myomectomy have been reported in a few select case studies [108]. In one report of 10 women pretreated with Cetrotide, 3 patients demonstrated no change or even an increase in fibroid volume based on MRI findings. The imaging for one woman did not permit a reliable interpretation of findings. In six women, however, MRI demonstrated a mean reduction of 31% in fibroid size following only 16 days of hormonal treatment [108]. Owing to the paucity of literature available, more studies are required to verify the outcomes of GnRH antagonists in the pretreatment of fibroids prior to myomectomy.

#### 7.1.3. Tranexamic Acid

Tranexamic acid (TXA) is an antifibrinolytic agent inhibiting the conversion of plasminogen into plasmin. Its use has become widespread in surgical procedures, postpartum bleeding, menorrhagia, and AUB. Use of TXA in the pretreatment of myomectomy, however, remains controversial. A recent meta-analysis concluded that total estimated blood loss was significantly lower in women who received TXA prior to myomectomy compared to controls, and this difference remained significant when intraoperative and postoperative blood loss was analyzed separately. Total operative time was significantly prolonged in controls; however, neither postoperative hematocrit and hemoglobin levels nor the number of women who received blood transfusions differed between the two groups [109]. A different meta-analysis also showed that mean intraoperative, postoperative, and total blood loss were significantly reduced in those who received pretreatment with TXA. Furthermore, mean postoperative hemoglobin and hematocrit levels were significantly higher in this group. While the mean length of hospital stay was significantly reduced in favor of TXA, there was no significant reduction in mean operative time and rate of blood transfusion [110]. In contrast, other studies have indicated that there is no significant decrease in intraoperative blood loss associated with intravenous TXA pretreatment. One randomized, double-blinded study on patients with large fibroids (one large fibroid >/= 10 cm, or intramural or broad ligament fibroid >/= 6 cm, or at least 5 fibroids), concluded that Intravenous administration of tranexamic acid in patients undergoing laparoscopic or robotic myomectomies was not associated with decreased blood loss [98]. Another trial also found no additional benefit of TXA on peri- and postoperative bleeding in gynecological surgery, concluding that it is not a useful adjunct in myomectomy [111]. Overall, there is currently no consensus regarding universal pretreatment with TXA prior to myomectomy and its administration should therefore be selective.

#### 7.1.4. Prostaglandins

Prostaglandins are commonly administered during postpartum hemorrhage owing to their profound effect of increasing myometrial tone. By the same token, vaginal deposition of prostaglandin one hour prior to myomectomy has been widely utilized with the aim of reducing intraoperative blood loss. One randomized control trial of 67 women found that intravaginal misoprostol administered prior to cases of conventional laparoscopic and LAM was associated with decreased estimated blood loss and a smaller decrease in postoperative hemoglobin compared to placebo [112]. In a Cochrane study, two randomized control trials with 89 women also showed a significant reduction in blood loss during myomectomy with intravaginal prostaglandin placement. There was no difference in the rate of blood transfusion [113]. Since the limited available data are based on low to moderate quality of evidence, interpretation of these results should be performed with caution. In general, prostaglandins are safe agents and their administration is largely regarded as reasonable, especially in cases where there is a concern of significant intraoperative bleeding.

#### 7.1.5. Other Medications

Other medications or protocols such as oxytocin, epinephrine, bupivacaine with epinephrine, vasopressin with misoprostol, ornipressin, and ascorbic acid have also been studied for preoperative treatment of fibroids. The currently published evidence remains inconclusive regarding the best management strategies to reduce blood loss during myomectomy. There remains a paucity of comparative studies of the different interventions and further randomized control trials are required to support the best recommended medical interventions for myomectomy [114,115].

#### 7.1.6. Peri-Cervical Tourniquet

Peri-cervical tourniquet is a mechanical technique commonly used in open myomectomy cases. Multiple studies have demonstrated its efficacy in reducing blood loss and the rate of blood transfusion [113,116,117]. Its use during minimally invasive myomectomy, however, is limited due to the difficulty in application of the tourniquet. The uterus usually requires exteriorization in order to open the broad ligament around the lower uterine segment bilaterally. Dr. MacKoul’s group reported their experience of laparoscopically applying a latex rubber tourniquet around the isthmus of the uterus in 969 women across a five-year period [88]. Laparoscopic defects were made in the broad ligament bilaterally, and the tourniquet was passed through them under direct visualization. A suprapubic incision was then extended 3–4 cm in either a transverse or vertical direction depending on the extent of exposure required. A small or medium wound retractor was placed, permitting the incision diameter to stretch to 6–8 cm. This is similar to a smaller open incision, and the tourniquet was then tightened through the incision. The surgeons achieved transient uterine artery blockade using the tourniquet in 62.8% of cases and permanent uterine artery ligation in 12.3%. In 24.9% of the cases, both techniques were used in combination. Although the group did not specify the indication for permanent ligation versus both techniques in combination, their results were highly satisfactory. The mean estimated blood loss was reported to be 192 mL (range 5–2000 mL) with only eight cases (0.7%) exceeding 1000 mL. The mean operative time was 70 min, with 80% of surgeries performed in under 90 min [88]. To the best of our knowledge, there are no groups besides Dr. MacKoul’s routinely practicing this technique in MIS. Its efficacy and feasibility therefore remain to be established and generalized by other surgeons.

#### 7.1.7. Uterine Artery Occlusion

The dissection and occlusion of the uterine arteries at different locations is well reported in both laparoscopic and robotic myomectomy [118,119,120,121,122]. A recent systematic review and meta-analysis of 25 studies (5 randomized control trials and 20 observational studies) including 2871 women undergoing surgical uterine artery blockade at the time of myomectomy found a statistically significant reduction in estimated blood loss, risk of blood transfusion, and decrease in postoperative hemoglobin compared to controls. These findings held for both laparoscopic and open approaches and the results remained consistent when randomized control trials and higher quality observational trials were analyzed separately [121]. The three main routes to access the uterine artery at its origin are the anterior, posterior, and medial approaches [118]. Whilst the posterior approach is commonly cited in the literature, there is a paucity of studies reporting its success rate, especially for cases of large uteri no longer confined within the pelvis. Surgeons should be familiar with all three approaches, given the potential variation and distortion in anatomy secondary to the location or size of fibroids. We recently reported our own experience of using uterine artery blockade via the anterior cul-de-sac. When the majority of fibroids are present within the uterine body, the anterior cul-de-sac usually remains accessible even if uterine size is extremely large and a posterior approach is not feasible. Uterine artery blockade can be performed either laparoscopically or through a mini-laparotomy, avoiding the need for a large transverse or even vertical incision [67]. As previously discussed, the operative time of laparoscopic myomectomy is relatively longer compared to abdominal surgery. With consideration of the associated risks and benefits, laparoscopic myomectomy is recommended for cases of fibroids less than three in number or 10 cm in size, otherwise laparotomy is regarded as more appropriate [123,124]. However, this criterion is highly arbitrary and the indication for laparoscopic myomectomy is largely based on surgeons’ experience and skills. Ultimately, the goal of laparoscopic myomectomy is to achieve the same outcome as an open approach. Minimizing intraoperative bleeding via transient uterine artery occlusion, in combination with other medical methods, is therefore a pivotal step to successfully performing laparoscopic myomectomy on large, multiple fibroids. It is a fundamental skill if a minimally invasive surgeon desires to perform advanced myomectomy, through conventional laparoscopy or a robotic platform.

Besides surgical occlusion, preoperative uterine artery embolization (PUAE) is a mechanical method also described in the literature. Published data, however, remain limited to individual reports or small case series [125,126,127,128]. PUAE can be carried out by an interventional radiologist in the hours to days prior to myomectomy. All studies thus far have demonstrated a significant reduction in blood loss for patients even with multiple large fibroids. There are, however, little data available on the long-term follow-up of these women, especially regarding fertility. One short-term six-month follow-up of 20 women who underwent PUAE prior to abdominal myomectomy found a mean decrease in uterine volume of 77.33% ± 14.25% and fibroid diameter of 46.45% ± 25.61%. Six of the women subsequently became pregnant, one of which had two separate pregnancies [126]. On the other hand, a different study investigated intrauterine synechiae in women following myomectomy with transient uterine artery ligation, PUAE or no preoperative treatment. Compared to those who underwent concurrent transient uterine ligation, both groups receiving PAUE or no pretreatment were found to have a higher incidence of synechiae, which were diagnosed and treated hysteroscopically. Following treatment of the synechiae, the pregnancy rate in all three groups was almost equal [127]. The effect of PUAE with myomectomy on fertility requires much further investigation, especially for women who desire future pregnancy.

### 7.2. Laparoscopic Suturing

There is no doubt that expertise in laparoscopic suturing is a critical prerequisite for successful laparoscopic myomectomy. There is evidence that simulation can affect outcomes, suggesting it is an effective way to improve skills in MIS and increase its utilization [129]. Firstly, it can improve the quality and safety of surgery. Secondly, simulation can decrease operating time, which leads to reduced costs and in turn increased utilization. The tactile gauging of simulation is, however, very different from that of real surgery. We recommend that gynecologic surgeons begin with training in laparoscopic vaginal cuff closure. Laparoscopic knot tying is also extremely beneficial for the hand-eye-brain coordination required to improve skills in suturing. Laparoscopic suturing during myomectomy should be as secure as it is in open surgery to achieve hemostasis and minimize the risk of hematoma formation. Myomectomy is a traumatic event to the myometrium and the healing process of this trauma is affected by several factors. Besides avoiding thermal coagulation for hemostasis, excessive suture also should be avoided, in that the polyglactine stitch has a reabsorbable time of 50–60 days. The foreign body reaction can delay the recovery of myometrium healing [130,131]. Closure of the myometrium can be in one to three layers, or more as needed, and utilization of a resorbable barbed suture is a recent innovation in laparoscopic myomectomy. According to a recent meta-analysis of eight studies, barbed suture significantly facilitates the procedure by reducing the time required for suturing and therefore total operative time, as well as reducing estimated blood loss, drop in hemoglobin, and rate of perioperative complications [132]. In animal models, barbed suture results in healing of uterine breaches comparable to traditional filaments [133]. There are presently no clinical studies comparing the myometrial healing process or rate of hematoma formation. Several studies have demonstrated similar pregnancy rates between women who underwent laparoscopic myomectomy using resorbable barbed suture compared to traditional filaments [134,135]. Concerns related to the use of barbed suture are those of adhesion formation. One recent study investigated this with second-look laparoscopy following laparoscopic myomectomy. The findings indicate that the incidence of postoperative adhesions with barbed suture for wound closure is similar to that with conventional suture [136]. Nevertheless, while advancements in suture materials and instruments are important, they do not replace the pertinence of surgeons’ skill. We firmly believe that surgical expertise is acquired through diligent and consistent practice and is a crucial trait of a successful and safe surgeon.

### 7.3. Pregnancy Complications

A major indication for myomectomy in reproductive-aged women is fertility preservation, and abdominal myomectomy is a well-established means to achieve this. Minimally invasive myomectomy should achieve similar if not better outcomes. On evaluation of the long-term fertility outcomes following robotic, laparoscopic, and open myomectomy, several studies have concluded that there are no significant differences between the groups [137,138,139]. Pregnancy rate after myomectomy has been reported to range from 22.5% to 70%, depending on patient age, the number and size of fibroids, and the degree of uterine cavity distortion [74,140,141]. In one retrospective cohort study, Dr. Shue et al. found that the number of fibroids removed during myomectomy had a significant effect on fertility. Women with over six fibroids removed were less likely to have a spontaneous pregnancy, more likely to require fertility treatment, and less likely to have a term live birth when compared to women with fewer than six fibroids removed [142]. Reproductive age is an independent factor affecting the rate of conception following myomectomy. In one observational prospective study, Dr. Tinelli et al. reported that the pregnancy rate is similar in women with or without a history of myomectomy if they are more than 40 years old [143]. Thus, it is significant to counsel patients regarding this factor if they are of advanced age and desire future fertility.

Myomectomy can lead to adverse pregnancy outcomes, regardless of approach, which must also feature in the counselling of patients before surgery. If multiple fibroids are removed, they are intramural or large, or the interval between myomectomy and pregnancy is short, the risk of obstetric and neonatal complications may increase [144]. Pregnancy after myomectomy may be associated with an increased risk of intrauterine adhesions, miscarriage, preterm birth, abnormal placentation, cesarean section, and uterine rupture [145,146]. Above all, uterine rupture during pregnancy is an established cause of stillbirth, perinatal hypoxic brain damage, cerebral palsy, and intrauterine fetal death [147]. In a large Korean multicenter case series, it was found that women with a history of fibroids or previous myomectomy had a significantly higher risk of cesarean section and placenta previa compared to women without a diagnosis of fibroids. The risk of uterine rupture was also significantly higher in women with a history of myomectomy. The incidence of uterine rupture was highest if delivery occurred within one year of myomectomy, and this risk decreased over time following surgery [148]. In a different study, women with a history of myomectomy also had a higher incidence of uterine rupture and placenta accreta [149]. The history of myomectomy increased the relative risk of uterine rupture 14-fold and was an independent risk factor for both placenta accreta and uterine rupture.

It is important to acknowledge that the absolute risk of uterine rupture following abdominal and laparoscopic myomectomy is low. Even in the large aforementioned Korean study, the occurrence of uterine rupture in such women was only 0.22% (22 of 9890 women) across an 11-year period [148]. A recent single-center study also reported that uterine rupture after laparoscopic myomectomy occurred in only 0.6% of pregnancies (3 of 523 women), miscarriage in 13%, preterm delivery in 10.4%, and full-term delivery in 76.7% [150]. One meta-analysis reported that the risk of uterine rupture was 0.4% following abdominal myomectomy, 1.2% following laparoscopic myomectomy, but neonatal mortality related to uterine rupture occurred in 33% of cases [151]. The incidence of uterine rupture may be a varying consequence of the size, type and location of fibroids removed, the suturing technique used during myomectomy, and the interval between the surgery and subsequent pregnancy [152,153]. One systematic review of eleven studies found the overall incidence of uterine rupture following myomectomy was just seven cases (0.93%). Two occurred during labor and the other five occurred within 36 weeks of gestation [154]. Thus, it is reasonable to consider offering and counselling patients on the option of trial of labor after myomectomy (TOLAM), similar to trial of labor after cesarean section (TOLAC). The incidence of uterine rupture in labor after cesarean section is reported to be 0.5–1% [155]. Based on the data currently available, there is little evidence to indicate which factors definitively lead to an increased risk of uterine rupture, such as fibroid type, size, endometrial cavity exposure, use of sutures, intramural hematomas, indentations, postoperative infection, and uterine fistulas [150]. On balance, the risks associated with vaginal delivery may outweigh those of cesarean section even in women who have previously undergone myomectomy. Individualized counselling with consideration of the available evidence and informed consent is therefore critical in the decision for route of delivery, which should be in a unit with the means to support and safely manage each patient’s choice [156].

Finally, it is notable that uterine rupture most frequently occurs during the late second or early third trimester of pregnancy. In Dr. Kim’s study of 14 cases of uterine rupture, there were no cases that occurred during labor [157]. A more recent meta-analysis reported the risk of uterine rupture after myomectomy was 0.75%, and this remained constant regardless of surgical technique or size of fibroids. Similarly, this group found that only 1 case of rupture occurred during labor, while the remaining 28 cases were before the onset of labor, and most (80%) occurred during the preterm period between 28 and 36 weeks of gestation [151]. It is therefore essential that obstetricians carefully follow women with a history of myomectomy during their prenatal care, given the potential maternal-fetal morbidity and mortality associated with uterine rupture [157]. Moreover, adequate counseling regarding the potential risk of uterine rupture during subsequent pregnancy should be provided to women of childbearing potential before any myomectomy procedure.

### 7.4. Uterine Sarcoma

Historically, laparoscopic myomectomy and hysterectomy were facilitated by power morcellation to aid with specimen retrieval. It was the incidence of dissemination of occult leiomyosarcoma with power morcellation in Brigham and Women’s Hospital in 2013 that first brought concerns regarding the safety of this technique to the attention of the public and medical societies. The FDA published its initial safety communication in April 2014, discouraging the use of power morcellation for the treatment of fibroids based on the risk of inadvertent spread of occult uterine malignancy [158,159]. The FDA estimates that the incidence of occult uterine sarcoma in women undergoing myomectomy or hysterectomy is 1 in 350 (0.28%), and unsuspected leiomyosarcoma is 1 in 498 (0.20%) [21]. Uterine sarcoma can be divided into three categories of uterine leiomyosarcoma, endometrial stromal sarcoma, and undifferentiated sarcoma. Uterine sarcomas are rare, with an estimated incidence of 0.36 per 100,000 woman-years [160]. Globally, the average age at diagnosis is 55 years [161].

The immediate impact of the FDA regulation was profound, and research has shown the changes to myomectomy in the 20 months before and after its publication. Results from one study indicate there was an 11% increase in the rate of abdominal myomectomy following the communication [162]. Another study also found a significant decrease in the proportion of minimally invasive hysterectomies and myomectomies performed during the eight months after. This study reported a decrease in the rate of minimally invasive hysterectomy by 8.7% and minimally invasive myomectomy by 19% [163]. Consequently, the rate of major and minor complications of hysterectomy appears to be increasing. According to one large-scale study by Dr. Multinu in 2018, investigating postoperative complications within 30 days among 25,571 women who underwent hysterectomy for fibroids, the rate of major complications following the FDA-issued safety warning on power morcellation increased from 1.9% to 2.4% (adjusted odds ratio [OR] 1.23; 95% CI, 1.04–1.47; *p* = 0.02) and minor complications from 2.7% to 3.3% (adjusted OR 1.21; 95% CI, 1.04–1.40; *p* = 0.01). In this subgroup of women, the incidence of open abdominal surgery increased from 37.2% to 43.0%, and the rate of MIS (total laparoscopic, laparoscopic supracervical, and laparoscopic-assisted vaginal hysterectomy) decreased from 56.1% to 49.7% (*p* < 0.001) [164]. Most gynecologists are of the position that the approval and disapproval of power morcellation by the same federal agency, the FDA, are lacking in scrutiny.

#### 7.4.1. Occult Uterine Sarcoma

There is a unanimous consensus that the iatrogenic spread of malignant tissue via power morcellation has serious clinical implications. Inadvertent dissemination of an occult uterine sarcoma by morcellation has been demonstrated to worsen prognosis with poorer disease-free and overall survival rates [165,166,167,168]. The argument for the prohibition of power morcellation, however, surrounds the true incidence of occult sarcoma and leiomyosarcoma. In one study from Turkey analyzing the outcomes of 6173 hysterectomies, the incidence of occult uterine leiomyosarcoma was calculated to be 0.08% [169]. A different study from Germany of 35,161 women undergoing laparoscopic myomectomy or hysterectomy found an incidence of leiomyosarcoma of 0.069% [170]. Other large-scale studies have reported similar rates of occult uterine sarcoma or leiomyosarcoma compared to the FDA statement [161,171,172,173]. Some groups, however, have reported an incidence higher than this, such as 0.303% for leiomyosarcoma [174] and 0.47% for uterine sarcoma [175] from two studies in China. It remains largely impossible to establish an international consensus regarding the true incidence of occult uterine sarcoma in cases of presumed fibroids. This is due to heterogeneity of the patient population, institute facilities, preoperative diagnosis, and workup performed.

#### 7.4.2. Risk Factors

Multiple studies have demonstrated that age is an independent factor in stratifying the risk of uterine sarcoma. In one retrospective cohort study, Brohl et al. predicted the occurrence of uterine sarcoma to range from approximately 5 cases per 500 for women aged 75 to 79 years to less than 1 case per 500 for women aged less than 30 years [171]. In another retrospective study of 24 unexpected cases of uterine sarcoma among 4478 women, the highest frequency of occult cancer (10 of 375, 2.6%) occurred at 51 to 60 years of age and the lowest (0 of 255) before 30 years [176]. Another study reported occult uterine sarcoma in 3 of 1398 women aged over 40 years, and no cases in the age group less than 40 [173]. Finally, one study found that the incidences of occult uterine leiomyosarcoma diagnosed following laparoscopic supracervical hysterectomy were 9.8, 10.7, and 33.4 per 10,000 for the age groups of 25 to 39, 40 to 49, and 50 to 64, respectively. The incidences following laparoscopic myomectomy were 0, 33.8, and 90.1 per 10,000 for the same age categories. Per the multivariate logistic regression analysis conducted, age was the only statistically significant risk factor for occult uterine leiomyosarcoma, with an age of 50 to 64 years associated with an increased risk of the disease [177]. Despite the lack of international consensus, we believe these data and the estimated incidence of occult uterine sarcoma and leiomyosarcoma established by the FDA can be used as a guide in clinical practice for patient counseling.

Black women have been established to exhibit an increased risk of uterine sarcoma and is associated with a twofold higher incidence of uterine leiomyosarcoma (but not other types of uterine sarcoma) compared to the risk for White women [178,179]. Postmenopausal status is also a risk factor for uterine sarcoma, with an incidence in women above 50 years of age more than four times greater than that in younger women (6.4 vs. 1.5 per 100,000) [178].

Rapid growth of a uterine mass, defined as an increase by 6 weeks gestational size within one year, as determined by physical examination by a gynecologist in a premenopausal woman, has previously been considered a predictive factor for uterine sarcoma [180]. Studies have, however, demonstrated that this may not be true. One prospective MRI study showed that benign fibroids can also undergo rapid growth. They reported 37 of 101 fibroids increased in volume by more than 30% over a three-month period, with the most rapid growth observed in fibroids under 5 cm in diameter [181]. A different study of 1332 women found a similar incidence of uterine sarcoma between those with a rapidly growing uterus (one of 371 women [0.27%]) and those without a rapidly growing uterus (two of 961 women [0.15%]) [180]. Nevertheless, a new or rapidly enlarging myometrial mass in postmenopausal women who are not on estrogen therapy should be regarded as highly suspicious for a uterine sarcoma until proven otherwise [182].

Other risk factors for leiomyosarcoma have been documented, including obesity, diabetes, postmenopausal bleeding, and tamoxifen use for more than five years. Additionally, the history of radiation therapy for childhood cancer, p53 gene mutations, and germ-line mutations in fumarate hydratase have been associated with the incidence of leiomyosarcoma [183]. Thus, a thorough review of patient history, endometrial biopsy, and imaging studies are critical for a gynecologist to be alerted to the possibility of uterine malignancy.

#### 7.4.3. Preoperative Evaluation

At present, there is no reliable preoperative diagnostic modality for uterine sarcoma or leiomyosarcoma and it is often diagnosed postoperatively following myomectomy, hysterectomy, or supracervical hysterectomy for presumed benign disease. Since uterine sarcoma is a malignant mesenchymal tumor, preoperative endometrial biopsy commonly yields unsatisfactory results. Yet, endometrial biopsy, rather than targeted biopsy of a suspicious fibroid, can be used to detect some instances of uterine sarcoma if the tumor extends into the endometrial cavity. This is more frequent with cases of endometrial stromal sarcoma than leiomyosarcoma. Biopsy can be performed either in the office using Pipelle or with dilation and curettage under sedation, with similar sensitivity demonstrated by both techniques. One Canadian study followed 68 women with leiomyosarcoma who underwent endometrial biopsy before surgery. The study reported a sensitivity for total diagnosis of malignancy was 52% and the sensitivity for leiomyosarcoma in particular was 35% [184]. Another study found that endometrial sampling successfully detected leiomyosarcoma preoperatively in 58.2% of women and that the use of hysteroscopy for sampling increased the detection rate threefold [185].

Although ultrasound is a highly sensitive and specific diagnostic modality for benign fibroids, its role in differentiating this from uterine sarcoma is minimal. From existing studies, leiomyosarcomas are associated with sonographic features including large size (over 8 cm), irregular borders, areas of cystic change or necrosis, increased central and peripheral vascularity, higher peak systolic velocity and lower resistive index compared to fibroids, and rapid growth as above [186,187,188]. The diagnostic utility of ultrasound is, however, uncertain. One small study of 111 women (6 with leiomyosarcoma, 7 with carcinosarcoma, and 98 with fibroids), for example, reported no evidence of differences in sonographic appearances, including in the mean resistive index of the tumors, between leiomyosarcoma and benign fibroids [188].

Among the imaging modalities available, MRI is associated with the highest accuracy for characterization of uterine masses prior to intervention [189]. This is due to properties of improved soft-tissue contrast, a larger field of view, diffusion sequences, and multiplanar sequences. The seminar study of Goto et al. in 2002 detailed the MRI features associated with leiomyosarcoma [190]. Over the two decades following its publication, imaging findings in multiple studies investigating leiomyosarcoma masses repeatedly describe intermediate to high signal intensity on T2-weighted imaging, irregular margins with the adjacent myometrium, high signal intensity on high–b value DWI, and corresponding low signal intensity on ADC maps. Peritoneal implants and enlarged lymph nodes on MRI are also considered to be findings predictive of leiomyosarcoma [189]. Whilst the MRI features of leiomyosarcoma may seem starkly different to those of fibroids, benign variants which represent approximately 1 in 100 fibroids may present with mimicking appearances. Fibroid variants include cellular leiomyoma, hemorrhagic or degenerating leiomyoma, lipoleiomyoma, myxoid leiomyoma, and uterine smooth muscle tumors of uncertain malignant potential (STUMP) [191]. Thus, the interpretation of MRI findings for leiomyosarcoma should be in combination with other clinical factors related to each patient. A protocol for imaging leiomyosarcoma and diagnosis with MRI alone has yet to be established.

The tumor marker associated with leiomyosarcoma, which has been extensively investigated, is the enzyme lactate dehydrogenase (LDH). LDH is considered non-specific, and total LDH and some of its isoenzymes appear to be altered in several malignancies of the genital tract [192]. Goto et al. reported both a high sensitivity and specificity of elevated total LDH and LDH3 for the diagnosis of leiomyosarcoma, in conjunction with contrast-enhanced dynamic MRI [190]. Other studies have found an increase in total LDH4 and LDH5 (muscle isoforms) and a relative decrease in LDH1 and LDH2 (heart isoforms) associated with leiyomyosarcoma [192,193]. Studies have also investigated the diagnostic value of an elevated LDH5:LDH1 ratio (greater than one) [193,194]. Recently, a group in Italy developed the Uterine Mass Magna Graecia (UMG) risk index, LDH3 þ (24/LDH1), by which elevated indices correlate with an increased likelihood of uterine sarcoma. Using a threshold of over 29 as an elevated index, they reported a diagnostic sensitivity of 100% and specificity of 99.6% [194]. One study applied to a US population also found that the cutoff of 29 had a high specificity of 91.1% for diagnostic exclusion of uterine sarcoma, although increased body mass index (BMI) was associated with higher UMG index values [195]. As per Dr. Spivak, it is also important to remain mindful of the implications of the positive (PPVs) and negative predictive values (NPVs) relating to screening tests. Since the former depends on the prevalence of disease and test specificity, the PPV of a positive test is poor for a rare disease such as uterine sarcoma unless test specificity approaches 100%. Assuming the prevalence of uterine sarcoma is between 1:255 and 1:580, a screening test with 99.6% specificity and 100% sensitivity has a PPV of between only 32.2% and 49.6%, respectively. On the other hand, if test specificity is 91.1%, the hypothetical PPV for the same disease falls to between 1.9% and 4.2% [195]. The value of LDH alone to diagnose or exclude leiomyosarcoma is therefore not reliable.

#### 7.4.4. High Clinical Vigilance of Uterine Sarcoma

So far, there are no universally accepted criteria to differentiate benign fibroids, sarcoma, or leiomyosarcoma. Clinicians can avoid missing suspicion of uterine malignancy by careful consideration of patient risk factors, instigating appropriate preoperative workup and counseling. In one large study from Norway, 26 of 4791 women were diagnosed with leiomyosarcoma. A total of 6 of these women were diagnosed preoperatively by means of endometrial biopsy and 14 women were managed with open hysterectomy and bilateral salpingooophorectomy owing to preoperative clinical suspicion of malignancy [196]. In a different study from Germany extending over 12 years, six cases were clinically judged as highly suspicious preoperatively and treated as if uterine malignancies. All six were subsequently confirmed as malignant on intraoperative frozen section. Only 1 of 2269 women underwent laparoscopic hysterectomy with morcellation, which was diagnosed as uterine sarcoma on pathology postoperatively [197]. Another study from Italy investigated the outcome of 5826 laparoscopic myomectomies, subtotal or total hysterectomies performed for presumed fibroids. This group reported a total of 48 cases with a final diagnosis of uterine sarcoma, the majority of which were recognized as suspicious for sarcoma preoperatively and so morcellation was avoided (*n* = 39; 81.3%). The occurrence of unexpected uterine sarcomas in this study was 0.1% (6 of 5826) and morcellation was utilized in the procedure of one of these women [198]. Though its occurrence cannot be excluded completely, these data strongly indicate the importance of retaining a high index of clinical suspicion and thorough preoperative workup to minimize the incidence of occult uterine sarcoma.

#### 7.4.5. Morcellation

Uterine leiomyosarcoma is the most common uterine mesenchymal malignancy, accounting for 1% of all uterine malignancies. The majority are present at Stage I, and clinical outcomes are highly variable. Although the comprehensive diagnosis and treatment of leiomyosarcoma is beyond the scope of this review, it has been extensively described in other high-quality articles [183,199,200,201,202]. The consensus relating to power morcellation with respect to occult uterine sarcoma remains unanimous in that uterine morcellation carries a risk of disseminating unexpected malignancy with an apparent increase in mortality and a negative impact on survival outcomes [165,203,204,205]. Furthermore, it is not uncommon to find the dissemination of benign fibroids into the peritoneal cavity following power morcellation. Dr. Kho and Dr. Nezhat published a case series of parasitic myomas in 12 women and concluded that most parasitic myomas may be iatrogenic secondary to surgical intervention, in particular if morcellation techniques were utilized [206]. Leiomyomatosis peritonealis disseminate (LPD) is rare; however, cases associated with power morcellation in laparoscopic myomectomy or hysterectomy have been reported in the literature [207,208,209]. A systematic review from Tulandi et al. reported that laparoscopic hysterectomy or myomectomy utilizing unconfined morcellation was also associated with an increased risk of iatrogenic endometriosis (1.4%), adenomyosis (0.57%), parasitic myoma (0.9%), and rarely LPD. Benign sequelae of uterine or fibroid morcellation were found in up to 1% of cases, and this is much higher than the prevalence of uterine sarcoma after morcellation. Whilst benign conditions carry less morbidity and mortality than malignancy, they are more common and may require further treatment, including re-operation [210]. Van der Meulen et al. reported an overall incidence of parasitic myoma of 0.12% to 0.95% in 69 cases from 44 studies reviewed, with a median time between surgery and diagnosis of 48 months (range one to 192 months) and a mean number of parasitic myomas of 2.9 ± 3.3 (range 1 to 16) [211]. Overall, we believe that power morcellation should be avoided in minimally invasive gynecologic surgery.

There is also controversy surrounding manual morcellation. One Japanese study investigated laparoscopic hysterectomy or myomectomy with scalpel morcellation and the occurrence of unexpected uterine sarcoma. A total of 15 women were diagnosed with uterine sarcoma postoperatively, of which 8 went on to receive adjuvant chemotherapy and 11 (78%) experienced reoccurrence [212]. A meta-analysis of four observational studies of women with uterine sarcoma concluded that morcellation (either scalpel or power method) was associated with a significantly higher recurrence and mortality rate compared to cases with no morcellation [213]. Other studies, however, have not demonstrated a clear detrimental effect of manual morcellation in occult uterine sarcoma. In a cohort study by Raine-Bennett et al., 125 cases of occult uterine sarcomas were identified among 34,728 women who underwent hysterectomy for fibroids. The unadjusted three-year probability of disease-free survival for no morcellation, power and non-power morcellation was reported to be 0.54, 0.19, and 0.51, respectively (*p* = 0.15), and overall survival 0.64, 0.75, and 0.68, respectively (*p* = 0.97) [167]. In a different study, where 17 of 18 women who underwent vaginal scalpel morcellation were found to have grade I uterine sarcoma, follow-up data at 17 to 54 months were available for 15 women all of whom had no evidence of disease [214].

Presently, recommendations from the American Association of Gynecologic Laparoscopists (AAGL) and ACOG are for contained morcellation with both manual or power methods [78]. Accordingly, three generations of morcellation bags have been developed for power morcellation. The most commonly utilized is the second-generation, so-called two-port or dual opening bag, and these are usually made of polyurethane or polyurethane-coated nylon fabric. The majority of bags are transparent, which increases the safety of the procedure as transparency allows for simultaneous visualization of the bag contents and surrounding abdominal or pelvic structures. Multiple studies have demonstrated the feasibility and safety of contained power morcellation [215]. Thus far, the largest study of this kind is a retrospective single-center analysis of 1120 women using the MorSafe^®^ thermoplastic polyurethane endobag [216]. Only two cases of small bag puncture were reported, with no other difficulties or complications related to bag manipulation. A total of 78.7% of specimens weighed more than 250 g (*n* = 881) and 9% more than 1000 g. The largest specimens, weighing 2933 g, 3183 g, and 4780 g, required two bags for complete morcellation and containment [216].

## 8. Conclusions

For the past 40 years, since the revolutionization of surgery instigated by Dr. Nezhat’s video-assisted endoscopy, myomectomy has been feasible through a minimally invasive approach. Whilst this has benefited hundreds of thousands of patients, the technique is not a straightforward process and remains an odyssey with numerous obstacles [19,217]. It is due to the sheer persistence and unwavering dedication of Dr. Nezhat and other pioneers that young generations of gynecologists can embark on the journey, acquiring the skills to embrace this innovative approach and harness the latest advancements in surgical technology.

New developments in the field continue to emerge, such as laparoscopic or transcervical fibroid radiofrequency ablation. The Acessa ProVu system, for example, is a laparoscopic ultrasound-guided approach to treating fibroids using radiofrequency energy which received FDA approval in 2012. Sonata, another technology centered on a transcervical approach to radiofrequency fibroid ablation, was approved by the FDA in 2018 [15]. We anticipate exciting data to be reported on these approaches in the forthcoming years [218]. High-intensity focused ultrasound (HIFU or MRgFUS) ablation of fibroids, which is more popularized in Eastern Asian countries, demonstrates promising results in terms of symptom treatment and obstetrics outcomes [219,220,221]. Following its advent in 1976 using urologic monopolar resectoscopes [222], hysteroscopic myomectomy is undergoing astounding developments in terms of instrumentation, further increasing the safety and efficiency of this procedure. The monopolar resectoscope has now almost completely been superseded by the bipolar resectoscope worldwide and morcellators, such as MyoSure^@^ and TrueClear^@^, are widely adopted in many centers, eliminating the use of thermal energy altogether.

The past twenty years have witnessed the advent and widespread application of the ERAS protocol (Enhanced Recovery After Surgery) in all surgical fields, including gynecology [223,224,225]. A minimally invasive approach, as the major component of the protocol, is recommended to be adopted by all surgeons whenever feasible and myomectomy should not be an exception. Due to the increasing societal tendency of delayed childbearing and availability of ART (Artificial Reproductive Technology), more women at a later reproductive age are opting for fertility-sparing surgery to manage symptomatic uterine fibroids. Thus, all such providers of women’s health care should equip and empower themselves to offer patients minimally invasive myomectomy (Table 1).

## 9. Future Implications

On reflection of the entire history and evolution of the surgical field, it is astonishing how rapidly minimally invasive surgery has replaced laparotomy in general [226]. Since the first laparoscopic myomectomy in 1979, extensive research has made this operation more feasible and safer. Pending inquiries do, however, persist and necessitate continued development, as well as a refinement of the ever-expanding body of the literature available.

1. Since excessive hemorrhage remains one of the main challenges of myomectomy in general, there is room for expansion regarding the outcomes of new medications, such as GnRH antagonists, as well as new mechanical approaches, such as our reported use of uterine artery blockade at the anterior cul-de-sac [67].
2. The association between fibroids and infertility remains inconclusive and controversial, particularly the effect of non-cavity-distorting type 3 fibroids. In addition to the location and size of fibroids, other factors should be considered in research, such as the timing of presentation/history, number of fibroids, fibroid degeneration, and symptoms.
3. Long-term follow-up and large-scale perspective research studies are crucial to assess the outcomes of minimally invasive myomectomy on patient quality of life, recurrence rate, infertility, and obstetric complications.
4. The training of a next generation of competent gynecological surgeons is also pivotal. The four-year general OBGYN residency training currently offered in the USA system may not be adequate and further post-graduate surgical fellowships including expertise in infertility may be needed. Recently, Dr. Camaran Nezhat established a two-year fellowship in infertility surgery, further exemplifying his extraordinary vision and commitment to addressing critical issues in the field.
5. Finally, though fundamental, basic research into the pathophysiology of uterine fibroids has received very minimal attention and investment. Recent studies highlight that the NIH funding for fibroid research, for example, is markedly low both in absolute terms and relative to disease burden [227,228].

In 2023, Dr. Nezhat proposed that surgery should be customizable, replicable, and democratized, which saw the dawn of the new age of digital surgery. This is a combination of robotics, data analytics, machine learning, artificial intelligence (AI), enhanced visualization, and instrumentation. In essence, digital surgery is a fusion of the human mind with AI [217]. The last five years of modern history have witnessed the innovation of AI, which can be categorized into four main developmental steps of reactive machines, limited memory machines, theory of mind, and self-awareness. Moving forward, we expect to experience the form of self-awareness to exhibit human levels of intelligence, eventually bypassing our own and becoming superhuman in capabilities [217]. Myomectomy, as a surgical procedure, will eventually be standardized and may even become non-surgical in the near future.

## Figures and Tables

**Table 1:** Highlights from this review.

Highlights
Minimally invasive myomectomy includes laparoscopic myomectomy, robotic myomectomy, laparoscopic-assisted abdominal myomectomy (LAM), and mini-laparotomy myomectomy.
Though inconclusive, prevailing studies support the efficacy of mechanical approaches such as peri-cervical tourniquet or uterine artery blockage for the management of intraoperative bleeding.
Expertise in laparoscopic and/or robotic suturing is a prerequisite for minimally invasive myomectomy.
The location and size of fibroids have a role in infertility. Types 0, 1, and 2 fibroids diminish fertility, while 6 and 7 do not. For type 3 fibroids, some studies indicate that a size greater than 4 cm may diminish fertility.
Overall, the occurrence of uterine rupture after myomectomy is no more than uterine rupture after c-section. Thus, TOLAM (Trial of Labor After Myomectomy) may be considered in most clinical situations.
There remains no definitive pre-operative diagnostic modality for uterine sarcoma, however, a high index of clinical suspicion and thorough work-up can significantly decrease the probability of occult diagnosis.
Contained morcellation is highly recommended due to various complications associated with non-containment in minimally invasive myomectomy.
